# Evaluation of *PIK3CA* mutations in advanced ER+/HER2-breast cancer in Portugal – U-PIK Project

**DOI:** 10.3389/fmolb.2023.1082915

**Published:** 2023-02-07

**Authors:** Ana Peixoto, Luís Cirnes, Ana Luísa Carvalho, Maria João Andrade, Maria José Brito, Paula Borralho, Nuno Coimbra, Pedro M. Borralho, Ana Sofia Carneiro, Lisandra Castro, Lurdes Correia, Maria Rita Dionísio, Carlos Faria, Paulo Figueiredo, Ana Gomes, Joana Paixão, Manuela Pinheiro, Hugo Prazeres, Joana Ribeiro, Natália Salgueiro, Fernando C. Schmitt, Fátima Silva, Ana Rita Silvestre, Ana Carla Sousa, Joana Almeida-Tavares, Manuel R. Teixeira, Saudade André, José Carlos Machado

**Affiliations:** ^1^ Serviço de Genética Laboratorial, Instituto Português de Oncologia do Porto Francisco Gentil (IPO Porto), Porto, Portugal; ^2^ IPATIMUP - Instituto de Patologia e Imunologia da Universidade do Porto, Porto, Portugal; ^3^ Serviço de Anatomia Patológica, Instituto Português de Oncologia de Lisboa Francisco Gentil (IPOLFG), Lisboa, Portugal; ^4^ Centro Hospitalar e Universitário de Coimbra, Coimbra, Portugal; ^5^ Unidade de Mama, Centro Clínico Champalimaud, Fundação Champalimaud, Lisboa, Portugal; ^6^ Serviço de Anatomia Patológica, Hospital CUF Descobertas, Lisboa, Portugal; ^7^ Faculdade de Medicina da Universidade de Lisboa, Lisboa, Portugal; ^8^ Serviço de Anatomia Patológica, Instituto Português de Oncologia do Porto Francisco Gentil (IPO Porto), Porto, Portugal; ^9^ Novartis Farma - Produtos Farmacêuticos, S.A., Porto Salvo, Portugal; ^10^ Serviço de Anatomia Patológica, Hospital de Santa Maria, Centro Hospitalar Universitário Lisboa Norte, Lisboa, Portugal; ^11^ Departamento de Genética Molecular, SYNLAB Genética Médica, S.A., Porto, Portugal; ^12^ Instituto de Anatomia Patológica, Lisboa, Portugal; ^13^ Serviço de Anatomia Patológica, IPO Coimbra, Coimbra, Portugal; ^14^ Faculdade de Medicina da Universidade do Porto, Porto, Portugal; ^15^ Escola Superior de Tecnologia da Saúde de Coimbra, Coimbra, Portugal; ^16^ Associação Portuguesa de Técnicas de Anatomia Patológica, Porto, Portugal; ^17^ GenoMed – Diagnósticos de Medicina Molecular, S.A., Lisboa, Portugal; ^18^ Instituto de Ciências Biomédicas Abel Salazar, Universidade do Porto, Porto, Portugal

**Keywords:** advanced breast cancer, ER+/HER2−, *PIK3CA* mutations, ring trial, validation, molecular pathology

## Abstract

**Background:** Around 40% of ER+/HER2-breast carcinomas (BC) present mutations in the *PIK3CA* gene. Assessment of *PIK3CA* mutational status is required to identify patients eligible for treatment with PI3Kα inhibitors, with alpelisib currently the only approved tyrosine kinase inhibitor in this setting. U-PIK project aimed to conduct a ring trial to validate and implement the *PIK3CA* mutation testing in several Portuguese centers, decentralizing it and optimizing its quality at national level.

**Methods:** Eight Tester centers selected two samples of patients with advanced ER+/HER2- BC and generated eight replicates of each (n = 16). *PIK3CA* mutational status was assessed in two rounds. Six centers used the cobas^®^ *PIK3CA* mutation test, and two used PCR and Sanger sequencing. In parallel, two reference centers (IPATIMUP and the Portuguese Institute of Oncology [IPO]-Porto) performed *PIK3CA* mutation testing by NGS in the two rounds. The quality of molecular reports describing the results was also assessed. Testing results and molecular reports were received and analyzed by U-PIK coordinators: IPATIMUP, IPO-Porto, and IPO-Lisboa.

**Results:** Overall, five centers achieved a concordance rate with NGS results (allele frequency [AF] ≥5%) of 100%, one of 94%, one of 93%, and one of 87.5%, considering the overall performance in the two testing rounds. NGS reassessment of discrepancies in the results of the methods used by the Tester centers and the reference centers identified one probable false positive and two mutations with low AF (1–3%, at the analytical sensitivity threshold), interpreted as subclonal variants with heterogeneous representation in the tissue sections processed by the respective centers. The analysis of molecular reports revealed the need to implement the use of appropriate sequence variant nomenclature with the identification of reference sequences (HGVS-nomenclature) and to state the tumor cell content in each sample.

**Conclusion:** The concordance rates between the method used by each tester center and NGS validate the use of the *PIK3CA* mutational status test performed at these centers in clinical practice in patients with advanced ER+/HER2- BC.

## Introduction

Female breast carcinoma surpassed lung carcinoma as the most commonly diagnosed cancer in 2020, with an estimated 2.3 million new cases (11.7%), and it was the fifth leading cause of cancer death (6.9%), according to GLOBOCAN (Sung et al., 2021). Over 70% of breast carcinomas are hormone receptor-positive (HR+) and human epidermal growth factor receptor 2-negative (HER2-), collectively designated as HR+/HER2-breast carcinomas (Setiawan et al., 2009; Howlader et al., 2014). Approximately 40% of estrogen receptor-positive (ER+)/HER2-breast carcinomas have mutations in the *PIK3CA* gene, leading to hyperactivation of the alpha isoform (p110α) of phosphatidylinositol-3-kinase (PI3K) (Cancer Genome Atlas Network, 2012; Goncalves et al., 2018; Mollon et al., 2018). The PI3K-protein kinase B (AKT)-mammalian target of rapamycin (mTOR) cascade is one of the major downstream signaling pathways in human cells (Fruman et al., 2017), and its deregulation has been implicated in breast cancer development and progression (Lee et al., 2015).

*PIK3CA* activating tumor mutations may occur in several domains of the p110α catalytic subunit, but mostly (≈80%) arise in four hotspots of the helical and kinase domains: E542K, E545K, H1047R, and H1047L (Zhao and Vogt, 2008; Kalinsky et al., 2009; Dogruluk et al., 2015). According to the Cancer Genome Atlas Network, *PIK3CA* mutations are more frequent in luminal A (45%) compared to luminal B (29%) subtypes, also occurring in basal-like (9%) and HER2-enriched (39%) subtypes (Cancer Genome Atlas Network, 2012). A recent study including a large number of metastatic breast carcinomas (n = 3871) confirmed these data, reporting the presence of *PIK3CA* mutations in 39% of HR+/HER2- and 37% of HER2-amplified tumors, besides 21% of triple-negative carcinomas (Albanell et al., 2019). The presence of *PIK3CA* mutations represents an independent adverse prognostic factor in breast carcinoma (Sobhani et al., 2018) and has been associated with more aggressive disease and poor outcomes in metastatic disease (Fitzgerald et al., 2019).

Despite the high incidence of *PIK3CA* mutations in breast carcinoma and their putative prognostic role, the therapeutic targeting of the *PIK3CA* gene has fallen short of expectations, with results from clinical trials with pan-PI3K inhibitors in solid tumors largely disappointing, namely due to unfavorable toxicity *versus* clinical benefit ratio (Hanker et al., 2019). The α-selective PI3K inhibitor alpelisib was the first PI3K inhibitor to demonstrate a progression-free survival (PFS) benefit in HR+/HER2-metastatic breast carcinoma with activating *PIK3CA* mutations. In the SOLAR-1 phase III clinical trial, the addition of alpelisib to fulvestrant in patients with disease recurrence/progression on or after prior aromatase inhibitor therapy resulted in an almost double median PFS compared to fulvestrant alone in the *PIK3CA*-mutated cohort (11.0 vs. 5.7 months, HR 0.65; 95% CI 0.50–0.85; *p* < 0.001), with an acceptable safety profile (André et al., 2019). Results from the SOLAR-1 trial led to the approval by the U.S. Food and Drug Administration (FDA) in May 2019 of alpelisib in combination with fulvestrant for postmenopausal women and men with HR+/HER2-, *PIK3CA*-mutated advanced or metastatic breast carcinoma, as detected by an FDA-approved test, following progression on or after an endocrine-based regimen (US Food & Drug Administration (FDA), 2019), and subsequently also to the approval by the European Medicines Agency (EMA) in July 2020 of alpelisib in combination with fulvestrant for postmenopausal women and men with HR+/HER2-, locally advanced or metastatic breast cancer with a *PIK3CA* mutation, after disease progression following endocrine therapy as monotherapy (European Medicines Agency, 2021). The application of Therascreen^®^ companion diagnostic test allowed the detection of 11 *PIK3CA* mutations in patients included in the SOLAR-1 trial: C420R, E542K, E545A, E545D [1635G>T only], E545G, E545K, Q546E, Q546R, H1047L, H1047R, and H1047Y (QIAGEN; André et al., 2019).

In Europe, companion diagnostic tests are not required for market authorization of targeted Oncology drugs, and the prescription of approved targeted therapeutic options relies on evidence of the required mutational status assessed through validated methods. The use of Therascreen^®^ is not widespread in Portugal, but multiple technologies and platforms are available for molecular testing, which can potentially be used to detect *PIK3CA* mutational status in HR+/HER2-breast carcinoma samples. These require local validation and implementation for clinical diagnostic purposes, similar to what has been previously done for several other actionable mutations in different tumor types (e.g., *EGFR* in lung cancer, *BRAF* in melanoma and lung cancer, etc.). Therefore, to enable local assessment of *PIK3CA* mutational status, hospitals and diagnostic centers should select, implement, and validate diagnostic methodologies that allow the molecular detection of all currently but also potentially actionable *PIK3CA* mutations.

Due to the absence of local validation and implementation, *PIK3CA* mutation testing was not routinely performed in HR+/HER2-breast carcinoma and remained largely unavailable in Portugal. This represents an unmet need since the assessment of *PIK3CA* mutational status is crucial for identifying patients eligible for treatment with PI3Kα inhibitors, particularly alpelisib, which is currently FDA- and EMA-approved in this clinical setting. The U-PIK project aimed to conduct a ring trial to validate and implement the *PIK3CA* mutation testing in multiple Portuguese centers, decentralizing the test and optimizing its quality at national level.

## Materials and methods

U-PIK was a research collaboration between 11 partners ─ 10 centers (seven hospitals and three private laboratories) from the North, Central, and Lisbon regions of Portugal, and Novartis ─ that consisted of a multicenter, inter-laboratory ring trial conducted between December 2020 and December 2021.

The study was conducted according to the guidelines of the Declaration of Helsinki. Ethical approval for the study was obtained from multiple boards: Ethics Committee of IPOLFG and Research Council, Unidade de Investigação Clínica, Instituto Português de Oncologia de Lisboa Francisco Gentil, EPE; Ethics Committee of Centro Hospitalar e Universitário de Coimbra; Ethics Committee of Centro Hospitalar e Universitário de Lisboa Norte and of Centro Académico de Medicina de Lisboa; Ethics Committee of Hospital CUF Descobertas; and Ethics Council and Ethics Council for Health of Fundação Champalimaud. Written informed consent for study participation or research purposes in the scope of the study was provided by participants or participants’ legal guardians/next of kin. In addition, ethical approval from other institutions was waived, as the study is in accordance with Article 19 ("DNA Banks and Other Biological Products") of Portuguese Law No. 12/2005 of 26 January ("Personal genetic information and health information"), which states that in the case of using retrospective samples of human origin or in special situations where the consent of subjects involved cannot be obtained due to the amount of data or subjects, their age, or another similar reason, the material and data can be processed but only for purposes of scientific research or epidemiological and statistical data collection (Law No. 12/2005; Kalokairinou et al., 2018).

Eight centers selected two samples of patients with advanced ER+/HER2-breast carcinoma from routine clinical practice and generated eight replicates of each, in a total of 16 samples. All samples were duly anonymized, and each sample was processed through an initial section stained with hematoxylin and eosin (H&E) followed by 24 sequential sections of 6 µm and a final section stained with H&E with the tumor area demarcated.

All samples were centralized at Institute of Molecular Pathology and Immunology of the University of Porto (IPATIMUP), which generated replicates of each, originating two sample sets with identical sample content comprising the overall group of Test samples. These two sample sets were sent to eight Tester centers, which assessed the *PIK3CA* mutational status in two rounds, 8 weeks apart, using their in-house methodology. Tables 1, 2 depict the characteristics of the ER+/HER2-breast carcinoma samples included in the analysis.

**TABLE 1:** Characteristics of the ER+/HER2-breast carcinoma samples included in the first *PIK3CA* testing round.

Sample	A	C	E	G	I	K	M	O
Patient age (years)	73	63	54	63	71	77	36	46
Tumor content	75%	80%	>20%	≈50%	30%	35%	40%	>20%
Necrosis	<1%	0%	>20%	≈5%	<5%	<1%	0%	0%
Histological type	IBC (NST)	IBC (NST)	IBC (NST)	IBC (NST)	IBC (NST)	IBC (NST)	IBC (NST)	IBC (NST)
Histological grade	G2	G2				G1	G3	G2
ER (%)	90–100	100	80	100	91–100	50	90	100
PR (%)	80–90	20	0	40	0	20	90	5
HER2 status	0	0	0	2+ DISH-	0	1+	0	2+ DISH-

All samples correspond to surgical specimens (4 primary carcinomas, 1 subcutaneous recurrence [C], and 3 metastases: 1 cervical lymph node [E], 1 hepatic [K], and 1 pleural [M]).

ER, estrogen receptor; PR, progesterone receptor HER2, human epidermal growth factor receptor 2; IBC (NST), invasive breast carcinoma of no special type; G, grade; DISH, dual *in situ* hybridization.

**TABLE 2:** Characteristics of ER+/HER2-breast carcinoma samples included in the second *PIK3CA* testing round.

Sample	B	D	F	H	J	L	N	P
Patient age (years)	42	63	30	59	89	63	55	48
Tumor content (%)	60	90	>20	≈40	40	70	60	>20
Necrosis (%)	<1	1	>10	0	<5	<1	0	0
Histological type	IBC (NST)	IBC (NST)	IBC (NST)	IBC (NST)	IBC (NST)	IBC (NST)	ILC	IBC (NST)
Histological grade	G2		G3			G2	G2	G2
ER (%)	60–70	100	15	95	91–100	95	95	100
PR (%)	90–100	100	0	95		30	0	90
HER2 status	0	0	0	1+	0	2+ DISH-	1+	0

All samples correspond to surgical specimens (7 primary carcinomas and 1 axillary lymph node recurrence [D]).

ER, estrogen receptor; PR, progesterone receptor HER2, human epidermal growth factor receptor 2; IBC (NST), invasive breast carcinoma of no special type; ILC, invasive lobular carcinoma; G, grade; DISH, dual *in situ* hybridization.

Tester centers were given 15 running days from the date of sample set reception to test *PIK3CA* mutational status and report back on results. As a study requirement, the in-house methodology of each Tester center had to allow the detection of mutations in at least exons 7, 9, and 20 of *PIK3CA* (corresponding to exons 8, 10, and 21 using the MANE Select transcript NM_006218.4), as per SOLAR-1 clinical trial criteria (ClinicalTrials.gov). Six Tester centers used the cobas^®^ *PIK3CA* Mutation Test, and two used PCR and Sanger sequencing (see Supplementary Material). In parallel, two reference centers (IPATIMUP and the Portuguese Institute of Oncology [IPO]-Porto) performed *PIK3CA* mutation testing by NGS in the two rounds (see Supplementary Material).

The concordance rate between the results of *PIK3CA* mutational status obtained by each Tester center and NGS was reported for both testing rounds. The concordance rate was also reported globally, considering all samples tested in both rounds at each Tester center. In addition, the quality of molecular reports describing *PIK3CA* mutation testing results was also assessed. The testing results from Tester sites and the molecular reports were received and analyzed by U-PIK coordinators: IPATIMUP, IPO-Porto, and IPO-Lisboa.

The original data presented in the study will be publicly available. The NGS data was submitted to the European Nucleotide Archive (ENA) at EMBL-EBI, under the project accession number:PRJEB58369 (https://www.ebi.ac.uk/ena).

## Results

### Results of the *PIK3CA* testing rounds

Of the eight participating Tester centers, six assessed the *PIK3CA* mutational status by cobas^®^ *PIK3CA* Mutation Test and two by PCR and Sanger sequencing. In the first testing round, all Testers obtained a 100% concordance between the results of *PIK3CA* mutational status retrieved by their in-house methodology and NGS. Only one sample processed by one Tester center gave an invalid result due to damage of the slide and unavailability of adequate DNA yield, which precluded reanalysis. In the second testing round, four Tester centers achieved 100% concordance, three 88% concordance, and one 63% concordance in the *PIK3CA* mutational status results obtained by their in-house methodology and NGS. Overall, considering the two testing rounds, five centers achieved a concordance rate with NGS results (AF ≥5%) of 100%, one of 94%, one of 93%, and one of 87.5%.

Table 3 shows the discordant results obtained in the two *PIK3CA* testing rounds.

**TABLE 3:** Discordant results obtained in the two *PIK3CA* testing rounds.

	*PIK3CA* mutational status				Center 1	Center 2	Center 3	Center 4	Center 5	Center 6	Center 7	Center 8
Testing method	NGS				COBAS	COBAS	COBAS	COBAS	PCR/Sanger	COBAS	PCR/Sanger	COBAS
Testing sample	HGVS coding region	HGVS protein	VAF IPO-Porto (%)	VAF IPATIMUP (%)								
I	c.3140A>G	p. (His1047Arg)	29	31	Concordant	Concordant	Concordant	Concordant	Concordant	Invalid	Concordant	Concordant
F (P21_225)	Wild type	Wild type	-	-	Concordant	Concordant	H1047X	Concordant	Concordant	Concordant	Concordant	Concordant
L	c.3140A>G	p. (His1047Arg)	33	33	Partially concordant: G1049R	Concordant	Partially concordant: G1049R	Concordant	Concordant	Concordant	Concordant	Concordant
N	Wild type	Wild type	-	-	Concordant	Concordant	H1047X	H1047X; C420R	Concordant	H1047X	Concordant	Concordant
**Concordance rate (%)**					**100**	**100**	**87.5**	**94**	**100**	**93**	**100**	**100**

HGVS, human genome variation society; NGS, next-generation sequencing; VAF, variant allele frequency.

Discordant results were due to discrepancies in the output of three samples (samples F, N, and L), two of which had remaining DNA available and were reanalyzed by NGS (including allele frequencies [AFs] <5%) at IPO-Porto and IPATIMUP reference centers (Tables 4, 5). In one sample (sample F), the H1047X mutation was identified by the Tester center but not by NGS (either in the initial or repeated analysis), being considered a false positive probably resulting from sample contamination. In sample N, the H1047X mutation was detected by three Tester centers but not by NGS analysis using a 5% AF cut-off. However, in NGS reanalysis and integrative genomics viewer (IGV) visualization, the c.3140A>G variant was identified with low AF (1–3%) in all three samples of the three Tester centers, suggesting that this is a low-frequency subclonal variant with heterogeneous representation in the tissue sections processed by the respective centers. Furthermore, in this sample, the c.1258T>C variant (C420R) was detected by one of the Tester centers in addition to the H1047X mutation. This variant was also detected in NGS reanalysis with an AF of 1% and may also correspond to a low-frequency subclonal variant with higher representation in the tissue section processed by that center. The third sample (sample L) for which discrepancies were found could not be reassessed by NGS due to DNA unavailability. In this sample, although the result obtained by the methodology of the Tester center was concordant with NGS for the presence of the H1047X mutation, a second mutation (G1049R) was detected by the cobas^®^ method used by that center (Tables 4, 5), which however was not validated by the Tester center using Sanger sequencing and might represent a known issue of cross-reactivity of the cobas^®^ kit for some variants.

**TABLE 4:** *PIK3CA* testing results of NGS reanalysis by IPO-Porto reference center.

Testing sample	Tester center	*PIK3CA* testing result by in-house methodology (COBAS)	*PIK3CA* testing result by NGS*	H1047X (c.3140A>G/T)	H1047X (c.3139C>T)	C420R (c.1258T>C)
N	Center 4	H1047X; C420R	wild type	Total count 7023 reads: A 6934; G 78 (**1.1%**); T 7 (≤**0.1%**)	Total count 7149 reads: C 7133; T 1 (**≤0.1%**)	Total count 2397 reads: T 2375; C 18 (**0.8%**)
N	Center 3	H1047X	wild type	Total count 9066 reads: A 8826; G 222 (**2.4%**); T 10 (≤**0.1%**)	Total count 9193 reads: C 9168; T 4 (**≤0.1%**)	
N	Center 6	H1047X	wild type	Total count 8104 reads: A 7996; G 96 (**1.2%**); T 3 **(≤0.1%**)	Total count 8273 reads: C 8225; T 25 (**0.3%**)	
F	Center 3	H1047X	wild type	Total count 7388 reads: A 7381; G 2 (**≤0.1%**); 2 (**≤0.1%**)	Total count 7507 reads: C 7485; T 5 (**≤0.1%**)	

ID, identification; IGV, integrative genomics viewer; NGS, next-generation sequencing.

*Only VAFs >3% were considered.

Bold values: % read of the total count reads.

**TABLE 5:** *PIK3CA* testing results of NGS reanalysis by IPATIMUP reference center.

Testing sample	Tester center	*PIK3CA* testing result by in-house methodology (COBAS)	*PIK3CA* testing result by NGS*	H1047X (c.3140A>G/T)	H1047X (c.3139C>T)	C420R (c.1258T>C)
N	Center 4	H1047X; C420R	wild type	Total count 4545 reads: A 4484; G 60 (**1.3%**); T 0 (0%)	Total count 4546 reads: C 4538; T 8 (≤0.1%)	Total count 3892 reads:T 3865; C 27 (1%)
N	Center 3	H1047X	wild type	Total count 2764 reads: A 2679; G 84 (**3.0%**); T 1 (**≤0.1%**)	Total count 3180 reads: C 3171; T 9 (≤0.1%)	
N	Center 6	H1047X	wild type	Total count 5182 reads: A 5126; G 56 (**1.1%**); T 0 (0%)	Total count 5181 reads: C 5174; T 7 (≤0.1%)	
F	Center 3	H1047X	wild type	Total count 3179 reads: A 3176; G 3 (**≤0.1%**); T 0 (0%)	Total count 2766 reads: C 2765; T 1 (≤0.1%)	

ID, identification; IGV, integrative genomics viewer; NGS, next-generation sequencing.

*Only VAFs >3% were considered.

Bold values: % read of the total count reads.

### Quality of molecular reports

The analysis of the molecular reports revealed inconsistencies in the description of sequence variants, highlighting the need to foster the use of the appropriate sequence variant nomenclature (HGVS-nomenclature), the international standard for reporting and exchanging information of DNA, RNA, and protein sequence variants in a consistent and unambiguous way (den Dunnen et al., 2016). It also showed the need to include the identification of reference sequences and state the tumor cell content of each sample in the report.

## Discussion

Despite significant accomplishments in the diagnosis and treatment of advanced breast cancer, it remains largely incurable. In recent years, several clinical studies have sought to identify novel molecular targets and predictive biomarkers that enable tailored management of these patients and improve outcomes. Within this approach, the pharmacologic targeting of *PIK3CA* mutations in ER+/HER2-advanced breast cancer has recently shown significant benefits after the development of endocrine therapy resistance. The U-PIK project aimed to evaluate the analytical performance of *PIK3CA* mutational status testing and contribute to its decentralized implementation in Portuguese centers. Within U-PIK, both testing results and molecular reports were analyzed by U-PIK coordinators IPATIMUP, IPO-Porto, and IPO-Lisboa.

The study found high concordance rates between the results obtained with the methodologies used in each tester center and NGS performed at the two reference centers (AF ≥5%):100% in five centers, and 94%, 93%, and 87.5% in one center each. These results validate the use of the *PIK3CA* mutational status test performed at those centers in the clinical diagnostics of patients with advanced ER+/HER2-breast carcinoma and enable these patients to be selected for an additional treatment option with PI3Kα inhibitors. The selective PI3Kα inhibitor alpelisib brought a renewed interest in *PIK3CA* as a predictive biomarker in HR+/HER2-disease, and the widespread use of the *PIK3CA* mutational status testing in the clinical practice will allow these patients to be identified and potentially benefit from this targeted agent, now available in their treatment armamentarium.

According to the literature, around 90% of *PIK3CA* mutations cluster in exons 9 and 20, and only ≈5–10% are found in other exons (Campbell et al., 2004; Samuels et al., 2004; Harlé et al., 2013; Arsenic et al., 2015; Martínez-Sáez et al., 2020). Exons 9 and 20 encode the helical and kinase domains of the *PIK3CA* gene, respectively, providing auto-inhibition of the tyrosine kinases, with mutations within these regions triggering the process of constant auto-phosphorylation and resulting in gain of function (Zhao and Vogt, 2008). Exon 4 has also been reported to be often altered in breast cancer, with the p.N345K mutation described with a 5.5% frequency in a recent analysis including 6338 breast cancer patients across 10 publicly available studies (Martínez-Sáez et al., 2020). Although this is a likely pathogenic variant according to the COSMIC and OncoKB databases (Chakravarty et al., 2017; Tate et al., 2019), its sensitivity to PI3K inhibitors has only been demonstrated in preclinical studies so far (Gymnopoulos et al., 2007; Dogruluk et al., 2015). In the present study, from the 16 specimens analyzed, 10 (62.5%) harbored *PIK3CA* mutations, predominantly found in exon 20 (n = 6, 37.5%) and exon 9 (n = 4, 25%), in agreement with most frequently reported *PIK3CA* mutations. Published studies show disparate results regarding the frequency of mutations in exon 9 and 20, with some also reporting that exon 20 is more frequently mutated than exon 9 (Li et al., 2006; Castaneda et al., 2014; Dirican et al., 2014; Loibl et al., 2014; Arsenic et al., 2015; Ahmad et al., 2016), and others showing otherwise (Campbell et al., 2004; Samuels et al., 2004). Nevertheless, the small sample size in this study should always be noted as a caveat and a limitation in the interpretation of the relative *PIK3CA* mutation frequency results in this study.

Four common mutations were identified in this small sample set, at codons 545 and 546 (E545K and Q546K) in the helical domain and at codon 1047 (H1047R and H1047L) in the kinase domain of the *PIK3CA* gene. All these alterations have been shown to respond to alpelisib (Verret et al., 2019; Anderson et al., 2020; Copur, 2020; Martínez-Sáez et al., 2020), although Q546K only in preclinical studies, where it has been associated with increased sensitivity to this agent (Mayer et al., 2017; Martínez-Sáez et al., 2020). In agreement with other reports, the four mutations observed in both exons were of missense type, with a single nucleotide change resulting in a different amino acid at the respective codons: nucleotide change c.1633G>A resulted in amino acid change p.E545K and nucleotide change c.1636C>A resulted in amino acid change p.Q546K at exon 9; nucleotide change c.3140A>G resulted in amino acid change p.H1047R and nucleotide change c.3140A>T resulted in amino acid change p.H1047L at exon 20 (Levine et al., 2005; Nosho et al., 2008; Ross et al., 2015; Ahmad et al., 2016). Both missense mutations E545K and Q546K were equally prevalent in exon 9 (50% each), while the H1047R missense mutation was more prevalent (83.3%) in exon 20 compared to the H1047L mutation (16.7%). These mutations were clustered within the mutational hotspot regions covering nucleotides 1633–1636 (n = 4) and 3140 (n = 6), in agreement with what has been reported by others (Nosho et al., 2008; Wang et al., 2015; Ahmad et al., 2016).

In addition to E545K, Q546K, H1047R, and H1047L, other less common *PIK3CA* mutations have been identified in the samples analyzed in this study. The exon 7 p.C420R mutation was found both in the assessment conducted by one Tester center and by NGS with an AF of 1%. This variant had also been previously reported by Corné et al. and Martínez-Sáez et al. with a frequency of 1.4% and 1.9%, respectively (Martínez-Sáez et al., 2020; Corné et al., 2021), and shown responsiveness to alpelisib in the SOLAR-1 trial (André et al., 2019). On the other hand, the *PIK3CA* exon 20 p.G1049R mutation was detected by the cobas^®^ method used by one Tester center, and had been previously reported in 0.7% of the samples analyzed in the study by Martínez-Sáez and colleagues (Martínez-Sáez et al., 2020). Although rare, this variant is likely pathogenic, with preclinical studies suggesting an increased sensitivity to alpelisib, similar to p.Q546K (Mayer et al., 2017) and p.E542K (Dogruluk et al., 2015) alterations. However, whenever this mutation is found in a tumor also presenting the H1047X mutation, the possibility of a false positive due to a known issue of cross-reactivity of the quantitative PCR technology should be considered.

The detection of *PIK3CA* mutations in liquid biopsy, although not in the scope of the U-PIK study, is also being explored in metastatic breast cancer setting with encouraging results (Corné et al., 2021; Galvano et al., 2022), and may play a role in identifying patients harboring *PIK3CA* and possibly additional mutations. An individual patient data meta-analysis recently ascertained the accuracy of circulating tumor DNA (ctDNA) for the detection of *PIK3CA* mutations, raising the possibility of replacing tissue for ctDNA tumor sampling in the future as a preferred strategy for metastatic breast cancer patients with low clinical compliance or inaccessible metastatic sites (Galvano et al., 2022). The latest ESO-ESMO guidelines have also acknowledged the assessment of plasma circulating free DNA as a good alternative to metastatic tumor analysis, as it may overcome the challenges (both for clinicians and patients) of obtaining metastatic tissue biopsies, and as an option for the selection of patients eligible for alpelisib (Cardoso et al., 2020). Furthermore, and further attesting to the relevance of identifying actionable *PIK3CA* mutations in breast cancer, major hot spot-activating missense *PIK3CA* mutations (E542K, E545K/A, H1047R/L) have a level IA of evidence for actionability according to the ESMO Scale for Clinical Actionability of molecular Targets (ESCAT; (Condorelli et al., 2019).

Overall, the results obtained in the U-PIK study validate the *PIK3CA* mutational status testing performed through cobas^®^ and PCR/Sanger sequencing (and NGS) at the eight Portuguese centers participating in this study and show the feasibility of adopting these methodologies as part of the clinical routine practice of patients with advanced ER+/HER2-breast carcinoma, decentralizing the analysis from reference laboratories.

## Data Availability

The datasets presented in this study can be found in online repositories. The names of the repository/repositories and accession number(s) can be accessed at: https://www.ebi.ac.uk/ena, PRJEB58369.
