# Corrigendum: Evaluation of *PIK3CA* mutations in advanced ER+/HER2-breast cancer in Portugal – U-PIK Project

**DOI:** 10.3389/fmolb.2024.1298522

**Published:** 2024-04-24

**Authors:** Ana Peixoto, Luís Cirnes, Ana Luísa Carvalho, Maria João Andrade, Maria José Brito, Paula Borralho, Nuno Coimbra, Pedro M. Borralho, Ana Sofia Carneiro, Lisandra Castro, Lurdes Correia, Maria Rita Dionísio, Carlos Faria, Paulo Figueiredo, Ana Gomes, Joana Paixão, Manuela Pinheiro, Hugo Prazeres, Joana Ribeiro, Natália Salgueiro, Fernando. C Schmitt, Fátima Silva, Ana Rita Silvestre, Ana Carla Sousa, Joana Almeida-Tavares, Manuel R. Teixeira, Saudade André, Jose Carlos Machado

**Affiliations:** ^1^ Serviço de Genética Laboratorial, Instituto Português de Oncologia do Porto Francisco Gentil (IPO Porto), Porto, Portugal; ^2^ IPATIMUP - Instituto de Patologia e Imunologia da Universidade do Porto, Porto, Portugal; ^3^ Serviço de Anatomia Patológica, Instituto Português de Oncologia de Lisboa Francisco Gentil (IPOLFG), Lisboa, Portugal; ^4^ Centro Hospitalar e Universitário de Coimbra, Coimbra, Portugal; ^5^ Unidade de Mama, Centro Clínico Champalimaud, Fundação Champalimaud, Lisboa, Portugal; ^6^ Serviço de Anatomia Patológica, Hospital CUF Descobertas, Lisboa, Portugal; ^7^ Faculdade de Medicina da Universidade de Lisboa, Lisboa, Portugal; ^8^ Serviço de Anatomia Patológica, Instituto Português de Oncologia do Porto Francisco Gentil (IPO Porto), Porto, Portugal; ^9^ Novartis Farma - Produtos Farmacêuticos, S.A., Porto Salvo, Portugal; ^10^ Serviço de Anatomia Patológica, Hospital de Santa Maria, Centro Hospitalar Universitário Lisboa Norte, Lisboa, Portugal; ^11^ Departamento de Genética Molecular, SYNLAB Genética Médica, S.A., Porto, Portugal; ^12^ Instituto de Anatomia Patológica, Lisboa, Portugal; ^13^ Serviço de Anatomia Patológica, IPO Coimbra, Coimbra, Portugal; ^14^ Faculdade de Medicina da Universidade do Porto, Porto, Portugal; ^15^ Escola Superior de Tecnologia da Saúde de Coimbra, Coimbra, Portugal; ^16^ Associação Portuguesa de Técnicas de Anatomia Patológica, Porto, Portugal; ^17^ GenoMed – Diagnósticos de Medicina Molecular, S.A., Lisboa, Portugal; ^18^ Instituto de Ciências Biomédicas Abel Salazar, Universidade do Porto, Porto, Portugal

**Keywords:** advanced breast cancer, ER+/HER2−, *PIK3CA* mutations, ring trial, validation, molecular pathology

In the published article, there was an error in the author list, and author Nuno Coimbra was erroneously excluded. The corrected author list appears below.

Ana Peixoto^1^, Luís Cirnes^2^, Ana Luísa Carvalho^3^, Maria João Andrade^4^, Maria José Brito^5^, Paula Borralho^6,7^, Nuno Coimbra^8^, Pedro M. Borralho^9^, Ana Sofia Carneiro^10^, Lisandra Castro^11^, Lurdes Correia^10,12^, Maria Rita Dionísio^9^, Carlos Faria^4^, Paulo Figueiredo^13^, Ana Gomes^4^, Joana Paixão^10^, Manuela Pinheiro^1^, Hugo Prazeres^13^, Joana Ribeiro^5^, Natália Salgueiro^11^, Fernando C. Schmitt^2,14^, Fátima Silva^4,15,16^, Ana Rita Silvestre^6^, Ana Carla Sousa^17^, Joana Almeida-Tavares^10^, Manuel R. Teixeira^1,18,^*, Saudade André^3,^* and José Carlos Machado^2,14,^*

Following this correction, the **Author contributions** section should also be corrected to include the contribution of author Nuno Coimbra as follows: “JCM, SA, MRT, and PMB conceived the U-PIK Project. JCM, SA, MRT, PMB, FCS, MJA, MJB, PB, MRD, HP, NS, ACS, JAT participated in the study design. AP, LCi, ALC, MJA, MJB, PB, NC, ASC, LCa, LCo, CF, PF, AG, JP, MP, HP, JR, NS, FCS, FS, ARS, ACS, JAT performed the experimental work involved in sample preparation, DNA extraction and PIK3CA testing and reporting. JCM, SA and MRT were responsible for analyzing PIK3CA testing data from U-PIK testers, for reporting anonymized PIK3CA testing performance back to U-PIK testers, and also for the analysis of molecular reports. JCM, SA, MRT, PMB and AP edited the manuscript, drafted and written with editorial assistance of JCS (Acknowledgements). All authors read and approved the final manuscript.”

In the published article, there was an error regarding the **affiliation** for Nuno Coimbra, who was erroneously excluded from the author list and should have affiliation *Serviço de Anatomia Patológica, Instituto Português de Oncologia do Porto Francisco Gentil (IPO Porto), Porto, Portugal*.

The authors apologize for these errors and state that these do not change the scientific conclusions of the article in any way. The original article has been updated.

