# Supplementary material for: Evaluation of PIK3CA mutations in advanced ER+/HER2-breast cancer in Portugal – U-PIK Project
Source: Front Mol Biosci. 2023 Feb 7;10:1082915. doi: 10.3389/fmolb.2023.1082915 (PMC9941536; doi:10.3389/fmolb.2023.1082915)
Supplement: Supplementary file 1 [file Table1.DOCX]

Supplementary Data

**Reference center 1/Tester 1 methodology**

*1A) Selected PIK3CA testing methodology:*

─ Real-time PCR: cobas® PIK3CA Mutation Test

─ Sanger sequencing: to validate positive variants identified by Real-time PCR and cobas® PIK3CA Mutation Test

─ Next generation Sequencing (comparator for *PIK3CA* testing concordance determination): minimum coverage >500X; Illumina NextSeq 550 platform, using Illumina`s TruSight Hereditary Cancer Panel.

1B) Methods for sample processing to generate U-PIK case samples:

*Pre-analytical phase*

─ Timely reception and sectioning of the surgical specimen (cold ischemia time <20 minutes).

─ Fixation of the specimen in an adequate volume of 10% Neutral buffered formalin (ratio formalin:tissue >10 up to 20:1). Ideal fixation time between 24h and 72h.

─ Annotation of cold ischemia and fixation times for all surgical specimens.

─ Inclusion of fragments into a paraffin block.

NOTE: Fragments can be collected prior to specimen fixation and should be no thicker than 3-4 mm and respect the ideal fixation times.

*Sample processing*

─ Sectioning of the selected paraffin block and placement of sections onto non-adherent glass slides properly numbered and identified, in the following order:

- #1 initial section for hematoxylin and eosin (H&E) staining
- #24 6 μm-thick sections
- #1 final section for hematoxylin and eosin (H&E) staining
- Delimitation of the tumor area of interest on H&E-stained slides

─ Tumor cellularity should be ≥20% tumor cells in the specimen for accurate test results.

1C) Methods for sample processing and *PIK3CA* testing:

*DNA extraction and quantification*

─ Macrodissection of the tumor area on 3 slides for each case.

─ DNA extraction using the cobas® DNA Sample Preparation Kit.

─ DNA quantification on NanoDrop spectrophotometer. Samples with poor DNA yield (<2 ng/μl) excluded (as per manufacturer`s protocol).

*PIK3CA* testing by Real-time PCR

─ *PIK3CA* mutational analysis test performed by Real-time PCR using cobas® PIK3CA Mutation Test (RUO).

─ Sanger sequencing to identify the variants detected. For variants for which it is not possible to perform this validation (due to the lower sensitivity of Sanger sequencing compared to real-time PCR), only Cobas result is reported.

*PIK3CA* testing by *Next-generation sequencing (NGS)*

─ Sample quantification in Qubit Fluorometer using 50-500 ng of DNA input for NGS.

─ Assessment of variants in *PIK3CA* gene coding and adjacent (+/- 12 bases) regions by next-generation sequencing (NGS; minimum coverage >500X; TruSight Hereditary Cancer Panel of Illumina on the NextSeq 550 platform).

─ Alignment and identification of variants with NextGene software. Annotation and screening of variants with Geneticist Assistant software. Descriptions of variants according to Human Genome Variation Society (HGVS) recommendations.

NOTE: variants with no clinical relevance (polymorphisms and/or variants classified in the literature and/or ClinVar as benign/probably benign) or present in the test samples with allelic fraction <5%, will not be reported. Additionally, variants of unknown significance with allelic fraction <10%, will also not be reported.

**Tester 2 methodology**

*2A) Selected PIK3CA testing methodology*

─ Real-time PCR: cobas® PIK3CA Mutation Test

2B) Methods for sample processing to generate U-PIK case samples:

*Pre-analytical phase*

─ Receipt and sectioning of the surgical specimen

─ Tissue fixation in 10% Neutral buffered formalin. Ideal fixation time between 24h and 96h

─ Specimen embedded into paraffin block

─ Sectioning of the selected paraffin block and placement of sections onto glass slides properly numbered and identified, in the following order:

- #1 initial section for hematoxylin and eosin (H&E) staining
- #24 6 μm-thick sections
- #1 final section for hematoxylin and eosin (H&E) staining
- Delimitation of the tumor area of interest on H&E-stained slides

2C) Methods for sample processing and *PIK3CA* testing:

*DNA extraction and quantification*

─ #3 6-μm sections.

─ Manual DNA extraction with cobas DNA sample preparation kit.

─ DNA quantification by spectrophotometry in NanoDrop spectrophotometer (Thermo Fisher). Samples with DNA yield <2 ng/μL excluded from the analysis.

*PIK3CA* testing by Real-time PCR

─ *PIK3CA* mutational analysis test performed by Real-time PCR using cobas® PIK3CA Mutation Test.

**Tester 3 methodology**

*3A) Selected PIK3CA testing methodology*

─ Real-time PCR: cobas® PIK3CA Mutation Test

*3*C) Methods for sample processing and *PIK3CA* testing:

*DNA extraction and quantification*

─ Slides obtained after taking 3 dewaxed sections from the FFPET stained by HE and reviewed by a pathologist to confirm tumor representation in the sample. Samples with less than 10% tumor tissue considered inadequate.

─ DNA extraction from 5 μm-thick dewaxed section from formalin-fixed, paraffin-embedded tumor tissue (FFPET) using cobas DNA sample preparation kit.

─ DNA quantification by spectrophotometry and adjustment to a fixed concentration to be added to real-time PCR reactions. Samples with DNA yield <2 ng/μL excluded from the analysis.

*PIK3CA* testing by Real-time PCR

─ *PIK3CA* mutational analysis test, amplification and detection of target region variants by real-time PCR using performed by Real-time PCR using cobas® PIK3CA Mutation Test, according to the manufacturer's instructions.

**Tester 4 methodology**

*4A) Selected PIK3CA testing methodology*

─ Real-time PCR: cobas® PIK3CA Mutation Test

4B) Methods for sample processing to generate U-PIK case samples:

*Sample processing*

─ Samples fixed in 4% buffered formaldehyde (VWR Chemicals) for approximately 37 hours at room temperature.

─ Sample processing in the Milestone PATHOS equipment - automatic microwave (MW) processor using the following protocol:

Buffered formaldehyde 4% - 2 hours from 30°C to 50°C (fixation)

Water rinse for 5 minutes

70% alcohol wash for 5 minutes

100% alcohol for 1 hour at 40°C with microwave (dehydration)

Isopropanol for 1 hour and 55 minutes at 55°C (clarification)

Isopropanol for 3 hours and 30 minutes from 50°C to 65°C (clarification)

Vaporization for 1 hour and 30 minutes in vacuum at 600 bars (without reagent)

Embedding in paraffin for 6 hours at 66°C from 600 to 100 bars

─ Selected samples with at least 10% tumor content per tissue area.

─ 6-μm sections cut and placed on glass slides, subsequently air dried.

4C) Methods for sample processing and *PIK3CA* testing:

*DNA extraction and quantification*

─ Sections deparaffinized with Xylene and placed in 100% alcohol. Slides air-dried afterwards.

─ DNA extraction using the cobas® DNA Sample Preparation Kit - 05985536190.

─ Tissue scraping from the slide into a 1.5-mL tube with lysis buffer containing proteinase K (PK); extra tube with a negative control also prepared.

─ Lysis of samples using lysis buffer, PK, and temperature. Tubes subsequently cooled down to room temperature.

─ Addition of DNA Paraffin Binding Buffer to the samples, followed by isopropanol for precipitation, using collection tubes and elution tubes. Three successive washes with wash buffer until final elution (DNA elution buffer), obtaining purified DNA for quantification and subsequent amplification.

─ DNA quantification in Nanodrop spectrophotometer (Thermo Fisher) through acquisition of two consistent readings and one negative control reading for each sample. DNA concentration should be ≥2μg/μL. Calculations to estimate the volume of extracted DNA to apply to the microplate.

*PIK3CA* testing by Real-time PCR

─ *PIK3CA* mutational analysis test performed by Real-time PCR using cobas® PIK3CA Mutation Test. ─ DNA amplification on COBASZ 480 instrument.

**Reference center 2/Tester 5 methodology**

*5A) Selected PIK3CA testing methodology*

─ Sanger sequencing: Amplification performed by PCR with primers designed for the intronic regions of the exons under study. Analysis performed using Mutation Surveyor software

─ Next generation Sequencing (comparator for *PIK3CA* testing concordance determination): ION Torrent with Oncomine FOCUS Assay.

5B) Methods for sample processing to generate U-PIK case samples:

*Pre-analytical phase*

─ Receipt of the surgical specimen.

─ If necessary, serial sectioning of the biological sample as follows:

#1 Initial 4-μm section for H&E staining

#24 6-μm sections, intended for molecular study

#1 Final 4-μm final section for H&E staining

─ Chosen block with at least 20% of neoplastic cells. Non-adherent, air-dried slides.

─ All cases reviewed by a pathologist responsible for selecting the tumor area(s) intended.

5C) Methods for sample processing and *PIK3CA* testing:

*DNA extraction and quantification*

─ Macrodissection of the tumor area, if suggested by the pathologist. If macrodissection not required, transferral of the entire sample to an Eppendorf tube.

─ DNA extraction on Promega Maxwell RSC 16 instrument using the DNA FFPE Kit.

─ DNA quantification on NanoDrop 2000. Samples with poor DNA yield (<2 ng/μl) excluded from the analysis.

*PIK3CA* testing by Real-time PCR

─ *PIK3CA* mutational analysis test performed by Routine Sanger sequencing through amplification of the sample by PCR with primers designed for the intronic region of the exons under study. Analysis performed using the Mutation Surveyor software.

*PIK3CA* testing by *Next-generation sequencing (NGS)*

─ NGS confirmation/validation in Ion Torrent technology using the Oncomine FOCUS Assay kit, which requires 15 ng of initial DNA. Minimum requirement of 500x vertical coverage to allow a sensitivity >99% for the detection of nucleotide substitutions with an allele fraction ≥5% in the assessed DNA. These detection limits refer to samples with more than 20% neoplastic cells. Data analysis on the Ion Reporter software. Only clinically relevant changes reported.

**Tester 6 methodology**

*6A) Selected PIK3CA testing methodology*

─ Real-time PCR: cobas® PIK3CA Mutation Test

6C) Methods for sample processing and *PIK3CA* testing:

*DNA extraction and quantification*

*Sample processing*

─ DNA isolation from formalin-fixed, paraffin-embedded samples (3 6-μm slide sections per sample).

─ Based on the image of H&E-stained sample, estimation of the percentage of tumor cells present in the previously delimited area of the sample by the pathologist.

─ Sample dewaxing and hydration through macrodissection of the tumor area, according to the image of H&E-stained sample and pathologist’s assessment.

─ Manual DNA extraction using Cobas® DNA Sample Preparation Kit according to the manufacturer's instructions.

─ Analysis of the yield and purity of extracted DNA by spectrophotometry using the NanoDrop 1000 spectrophotometer according to the manufacturer's instructions. Only samples with concentration ≥ 2 ng/μL and absorbance ratios at 260/230 nm of 1.8-2.2 and at 260/280 nm of 1.8 included.

*PIK3CA* testing by Real-time PCR

─ *PIK3CA* mutational analysis test performed in cobas®Z480 instrument by real-time PCR using cobas® PIK3CA Mutation Test kit, according to the manufacturer's instructions and using negative and positive controls.

**Tester 7 methodology**

*7A) Selected PIK3CA testing methodology*

─ PCR + Sanger sequencing

7C) Methods for sample processing and *PIK3CA* testing:

*DNA extraction and quantification*

─ DNA extraction from paraffin-embedded tumor tissue with QIAamp DNA FFPE tissue kit (Qiagen) using the Qiacube automated extractor (Qiagen).

─ DNA quantification and quality assessment by spectrophotometry using NanoDrop™ One Microvolume UV-Vis (Thermo Fisher). DNA samples with a concentration <15ng/μl excluded.

─ DNA amplification by PCR using specific primers for exons 7, 9, and 20 of the *PIK3CA* gene.

*PIK3CA* testing by Real-time PCR+Sanger Sequencing

─ Sanger sequencing of amplification products on ABI3500 Genetic Analyser (Thermo Fisher).

─ Analysis of results using the Mutation Survey program, with full verification of the sequences obtained by the analyst.

─ Reporting of the variants identified.

This analysis assumes that the tumor sample is evaluated by a pathologist and has at least 20% tumor cells.

**Tester 8 methodology**

*8A) Selected PIK3CA testing methodology*

─ Real-time PCR: cobas® PIK3CA Mutation Test

8C) Methods for sample processing and *PIK3CA* testing:

*DNA extraction and quantification*

─ Histopathological selection of the specimen for molecular study through evaluation of the representative H&E-stained slide by a pathologist.

─ If applicable, macrodissection for enrichment of the sample to be tested.

─ Samples decalcified, without tumor tissue, or with insufficient tissue for testing (i.e., <10% neoplastic cells) excluded.

─ Manual DNA extraction from the selected tissue with cobas® DNA Sample Preparation Kit, according to the manufacturer's instructions.

─ DNA quantification by spectrophotometry in NanoDrop spectrophotometer (Thermo Fisher). Samples with a DNA yield <2 ng/μL excluded from the analysis.

*PIK3CA* testing by Real-time PCR

─ Amplification and detection of the specific target region variants by real-time PCR using cobas® *PIK3CA* Mutation Test kit according to the manufacturer's instructions.
